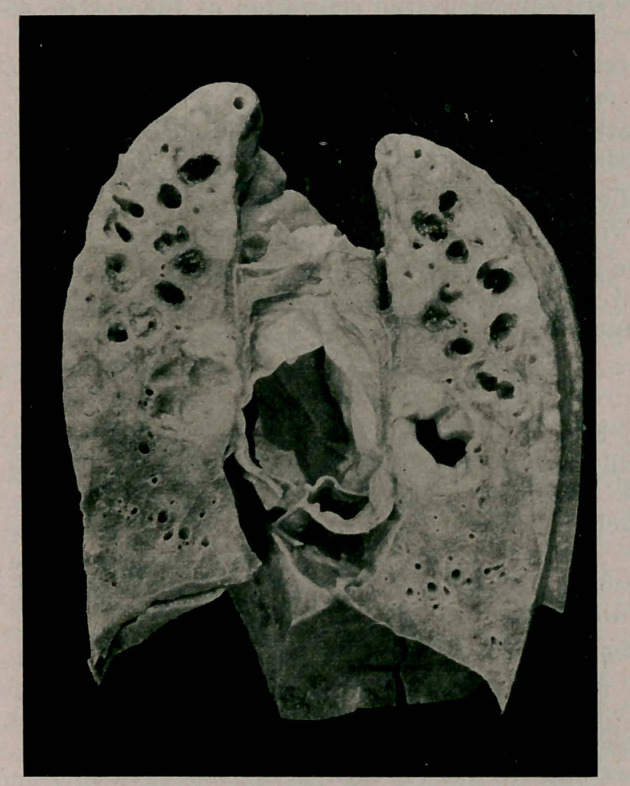# Hemoptysis in Infants

**Published:** 1914-07

**Authors:** Carl G. Leo-Wolf

**Affiliations:** Buffalo, N. Y.; 481 Franklin Street


### BUFFALO MEDICAL JOURNAL

Yearly Volume 69 JULY, 1914 No. 12

### ORIGINAL ARTICLES

The right is reserved to decline papers not dealing with practical medical and surgical subjects, and such as might offend or fail to interest readers. Contributors are solely responsible for opinions, methods of expression and revision of proof.

### Hemoptysis In Infants With Report of One Case.* (*)

By CARL G. LEO-WOLF, M. D.,

Of Buffalo, N. Y.

In the following lines I wish to report a case which I had the good fortune to observe at the Stefánia Children's Hospital in Budapest early this year, and which the director of this institution, Professor Dr. J. von Bókay, considered to be of sufficient interest to warrant its publication; and I am glad to have this opportunity to express to Prof. von Bókay my deep appreciation of the encouragement he has given me in my work, as well as for many a personal kindness conferred upon me.

Béla N., 11 months old, was received into the internal ward February 3rd, 1914, with the following history:

The child had been sick for one week; it had been languid, and in the beginning of its sickness it had several attacks of vomiting; since the day before it arrived at the hospital it had been unconscious and presented opisthotonus. No tubercular history in the patient's family. Immediately preceding the patient's reception into the Stefánia Hospital lumbar puncture had been performed at another children's hospital.

---

* Read before the Section of Pathology of the Buffalo Academy of Medicine, May 19th, 1914.

(*) From the Stefánia Children's Hospital Pediatric Clinic of the University of Budapest, Hungary (Prof. Dr. J. v. Bókay Director).

(In this connection I want to state that in Budapest lumbar puncture is performed systematically in the ambulatory service in all cases of meningitis (1) whatever their etiology, also in spasmophilia and in chronic hydrocephalus (2) ; I had occasion to see many of these cases during my two months stay there, and I have never seen any bad results from this procedure, but on the contrary I have seen a number of cases of diplococcus meningitis cured by it, and the records of the Stéfania Hospital show the recovery of two cases of tubercular meningitis with positive bacillary findings under this measure). (3).

To return to our case:

The status of the child at its reception into the hospital was as follows:

Weight 7800g.

Moderate rickets; caput quadratum, large fontanelle the size of a quarter dollar, thickening at the osteochondral lines, thickening of the epiphyses; the child has seven incisors.

Over the lungs nothing abnormal is to be detected by percussion, auscultation reveals a few râles.

Heart normal.

Abdomen retracted and no resistance can be felt.

Trousseau pronounced; pupils react readily; patellar reflexes exaggerated; the child lies without moving, the eyes are in fixation, there is rigidity of the neck.

Temperature 99.3.

February 4th: Temperature: 100.2 and 99.6; pulse 168, no change in the general condition; facial paralysis on the left side; at lumbar puncture about 15cc. of liquor is evacuated under moderate pressure in which fibrin coagulates on standing; cytological examination of it shows lymphocytes and several polynuclear leucocytes; a number of tubercle bacilli were also found; v. Pirquet reaction positive.

February 5th: Temperature 102.5 and 100.4, pulse 166; stadium paralyticum pronounced; **frequent bleeding from mouth and nose, the blood is bright red and foamy; tarry stools.**

February 6th: Temperature 104.0; frequent attacks of eclampsia; intermittant inspirations; Biot's respiration; death ensues at 10 a. m.

Post mortem was performed 24 hours after death by the prosector, Dr. F. Orsós, to whom I am greatly indebted for the anatomical report as well as the loan of the specimen.

I shall anticipate the anatomical diagnosis as I intend to go into details only as far as the lungs are concerned.

The post mortem diagnosis was:

Tubercular basilar meningitis with acute internal hydrocephalus of considerable amount; peribronchitis and caseous ulcerating pneumonia of the left upper lobe with rather small and confluent cavities; casous breaking down of the bronchial lymphatic nodes which are in size up to that of an almond; bronchiectases; pulmorrhagia; acid pulmomalacia of the right upper lobe; disseminated tubercules of the spleen and liver; inanition.

**The Left Lung** is free from adhesions except at the hilus, where it is solidly adherent to the mediastinum owing to the caseous degeneration of the bronchial lymphnodes. Its pleura is in general smooth, only over the outer surface of the upper lobe do we find it thickened in some places. The whole upper lobe feels somewhat dense and in some spots nodular; almost everywhere do we see disseminated yellowish tubercles under the pleura. In the lower lobe we can see some subpleural tubercles only in the angular region.

The lungs were injected through the bronchi with formalin without any pressure after the method of Dr. Orsós, and were preserved in the same solution.

After fixation the whole left lung was divided by parallel frontal cuts into slices of the thickness of about 0.5 cm. The cut surfaces of the upper lobe now presented the following picture:

The lung tissue in general is thickened all through the upper lobe, it does not contain any air, is remarkably anemic, moderately translucent and juicy. In the regions of some of the lobules we find in this lung tissue which is translucent and infiltrated with a gelatinous substance an exceedingly fine punctation which corresponds to the alveoli in size and is bright yellow in color—a pneumonia desquamativa. One part of the apex corresponding in size to that of a walnut is almost normal and contains air.

On the surface of a cut made through the middle of the hilus we observe the following interesting picture:

At the hilus we notice closely adjacent to the upper lobe a lymphnode, the cut surface of which is the size of a bean and which is entirely caseous. Between the branches of the blood vessels and the bronchial tubes, but seemingly inside the lung tissue, we perceive a second lymph node which is the size of a cherry pit, and a third one which is caseous and the size of a filbert. The two latter lymph nodes are surrounded by a fairly solid layer of connective tissue which contains the large blood vessels and the bronchial tubes which are compressed and therefore flattened.

In the center of the second lymph node, the one which is the size of a filbert, we see an isolated part which looks like a sequestrum and is the size of a pea, this is surrounded by a space which is filled with fairly fresh dark red blood, and its whole cut surface shows a pink imbibition.

In the lateral part of this lymphnode we find the main branch of the bronchus for the upper lobe, but this is compressed so that its lumen is a mere slit.

Where this lymph node and the bronchus lie close together we find in the outer layer of the lymph node as well as in the wall of the bronchus a discontinuity to the extent of two or three mm., so that the space surrounding the caseous sequester and the lumen of the bronchus communicate. The channel of this perforation, for this is what this is, is filled with a blood coagulum which contains a fine-grained detritus.

On the same cut surface we see in the center of the lobe the diameters of several bronchi the lumina of which have been enlarged by caseous degeneration.

In the anterior part of the lobe we find the other end of the lymph node which we described as being the size of a cherry pit in the cut we have just spoken of, but here it is much larger and is found to contain a focus of serous softening equal in size to that of a bean.

In the tissue of the lobe itself we find a number of cavernous bronchiectases which are either simple dilatations and have smooth walls, or are the results of caseation; some of these contain a little aspirated and coagulated blood. The infiltrated tissue between these bronchiectases contains numerous tubercles.

A very careful scrutiny of all cut surfaces reveals neither in the bronchi nor in the microscopical blood vessels any perforation except through the wall of the large bronchus.

In this case the hemoptysis was evidently due to an erosion of the blood vessels of the bronchial wall.

In the lower lobe of the left lung we find only a diffuse and quite recent thickening of some disseminated tubercles in the angular region; the whole lobe does, however, contain air and it is free from any older pathological changes.

**The Right Lung** showed only some recent softened spots which were caused by the aspiration of the gastric secretion, and further a moderate edema of the lower lobe.

The lymph nodes of the right hilus were also found to be in caseous degeneration.

**In Both Lungs** only a few disseminated lobules were found to contain a small amount of aspirated blood.

**The Left Main Bronchus and the Trachea** contained blood mixed with mucus.

**In the Stomach** were found about 50cc. of a mass consisting of blood mixed with mucus, and the same was found in the **Duodenum.**

I have given such a detailed description of this case for two reasons:

**First,** because the bronchietases are so extensive that we can hardly regard these as a secondary condition, but we must consider these to be the primary affection which has also, most likely, furnished the disposition for the tubercular infection; we see here a localization and a picture of the origin of tuberculosis in an infant such as we are accustomed to find it in adults only.

My **Second** and principal reason for publishing this case is the rare occurrence of hemoptoe in children and especially in infants which is conceded by all writers on this subject.

Let me quote briefly from some of the text books:

Barthez and Rilliett (4) have seen some cases of hemoptysis in tubercular children, this was almost always terminal and very copious.

Biedert (5) says: Hemoptysis is very rare in childhood, especially in young children, though it may occur in very young children. The youngest children on record to die from hemoptysis were one a little over one year old reported by Wyss (6) in 1878, and one of 20 months reported by Steffen (7) in 1869.

Brown (8) states that hemoptysis may be said to be infrequent in children under 10. Certainly it is very rare at the onset. It is usually small in amount. The terminal hemoptysis common in the adult, but rare in children, results from the rupture of an aneurism in a small cavity or the erosion of a branch of the pulmonary artery.

Cornet (9) writes that age plays an important part in the occurrence of hemoptoe, inasmuch as it is rare in subjects before puberty, especially before the second dentition.

Dennig (10) states that hemorrhages in considerable amounts, such as we observe these so frequently in adults, are very rare in childhood; he has not been able to find a single case in the records of his institution.

D'Espine and Picot (11) remark that hemoptoe is usually absent in children.

According to Finkelstein (12) hemoptoe is exceedingly rare in infancy.

In Hennig (13) we find that tubercular children do not spit up or cough up blood in the beginning of the disease, the same as do adults, but a severe hemoptoe may come at the end.

Henoch (14) found hemoptysis rare in children before the second dentition, though he will not go as far as Rilliet and Barthez who had stated that they had never seen it occur before the sixth year. He had seen blood in the sputa of at least a dozen children under five, but only in three of these in considerable amounts.

Holt (15) has personally never met with a case of hemoptysis under five years, and he declares it to be a rare symptom, but not unknown even in young children. He quotes: "Herz in 247 clinical cases of tuberculosis in children records 8 of hemoptysis, four of them under five years, and the youngest only eighteen months old. The records of 131 autopsies on tuberculous children in the Pendlebury Hospital show that hemoptysis was found four times a cause of death; two of these patients were under five years, and one was only twelve months old."

Hutinel (16) writes: Hemoptysis is rare in the early period of life, those cases which were reported were mostly terminal and profuse ones, and were due to changes in a large branche of the pulmonary artery caused by ulcerations from lymph nodes. The blood may come from other organs, such as the nose, pharynx, larynx, and even the ear. When it is surely proven that the blood came from the lungs, then one should remember that Trousseau has observed hemoptysis in pertussis, Bouchut (38) in pneumonia, Rilliet and Barthez in pulmonary gangrene. Mantel (52) has called attention to the fact that slight hemorrhages may be overlooked because the blood is swallowed.

v. Huttenbrenner (17) remarks: Hemorrhages from the lungs are very rarely observed in childhood in spite of the frequency of the destructive processes in phthisis; but if they should occur, they will be quite profuse.

Jacobi (18) out of his large experience does not remember more than half a dozen cases in children, except those which took place in violent attacks of whooping cough. Only one of his cases of hemoptoe in phthisis was three years old. Blood is not a frequent admixture in the expectoration of phthisical children; now and then it is met with, but profuse hemorrhages are rare in children.

Koplik (19) says: Hemoptysis is very rare in infants. He has seen, however, several cases in children of more than six years of age.

V. Pirquet (20) writes that hemoptoe, which is frequently the first sign of phthisis in adults, is very rare in children.

Rotch (21) finds hemoptysis rare in infants and in young children.

Steiner (22) makes the same statement and quotes one case of his own observation in a child of three years of age.

Sticker (23) states that hemoptoe before the seventh year is almost unheard of, but from the literature may be collected a number of such cases in early life.

Thomson (24) remarks that hemoptoe is comparatively rare in childhood. It does not occur, as in adults, as an early symptom of pulmonary phthisis, and is only rarely met with in the late stages of the disease.

Unger (25) writes that in children the sputa are only rarely streaked with blood; still rarer are hemoptoe or profuse hemorrhages.

West (26) observed almost always absence of hemoptysis in the beginning of phthisis in children, and relatively rarely in its course.

Young (27) remarks: Hemoptysis may occur in the form of streaking; but profuse hemorrhage, though it does occur, is extremely rare.

Zuber (28) speaks only of the profuse hemorrhages due to rupture of an aneurism, which were first described by Rasmussen (29), or to the rupture of one of the vascular bridges which are found to traverse the cavities in children.

Other writers of text books on diseases of children whom I have consulted, either do not mention hemoptysis or only quote a few cases of their own experience, or recite again those described by others.

Hinz (30) gives a careful description of a case of hemoptysis in an infant of twelve weeks together with the report of the obduction; he also quotes 44 other cases which he has collected from the literature. I shall give in my Bibliography appended to this paper the different authors who have published cases of hemoptoe in children, most of whom are quoted by Hinz, but I have tried wherever possible to complete his data so as to make it easier in the future to find these. (31 to 62).

Undoubtedly other cases of hemoptoe in children may be found in the literature, such as those reported by Ausset (63), Bierbaum (64), Gangoux et Maillet (65), but I trust that I have adduced sufficient proof of the comparative rarity of this condition in children and especially in infants.

To recapitulate: We find the most interesting points in this case to be the congenital bronchiectases which most likely caused in this case a form of tuberculosis not usually found in children; further that the extensive tuberculosis of the left upper lobe and the considerable tuberculosis of the bronchial lymph nodes gave rise to hardly any symptoms during life, though they were naturally obscured by the meningeal symptoms; and finally the profuse hemoptysis which, as we were able to prove, came from the perforation of a caseous hilus gland into a bronchus of the first degree.

481 Franklin Street.

### BIBLIOGRAPHY:

1. v. Bókay, J.: Der Wert der systematischen Lumbalpunktion in der Behandlung der Cerebrospinalmeningitis. Deutsche medizinische Wochenschrift 1907, No. 47.

2. v. Bókay, J.: Ueber den Wert der systematischen Lumbalpunktion bei der Behandlung des Hydrocephalus chronicus internus bei Kindern. Jahrbuch für Kinderheilkunde 1903, vol. 57, page 229.

3. v. Bókay, J.: To appear soon in the Deutsche Medizinische Wochenschrift.

4. Barthez & Rilliet: Handbuch der Kinderkrankheiten, Deutsch von Hagen Leipsic 1855.

5. Biedert, Ph.: Lehrbuch der Kinderkrankheiten, Stuttgart 1902, page 420.

6. Wyss, O.: Die Lungenschwindsucht, in Gerhardts Handbuch der Kinderkrankheiten, Tübingen 1878, vol. III, part 2, page 807.

7. Steffen, A.: Klinik der Kinderkrankheiten, 1869, vol II.

8. Brown, D.: Tuberculosis in Starr, L.: An American Textbook of the diseases of Children, 2nd edition, Philadelphia 1900, page 295.

9. Cornet, G.: Die Tuberculose, in Nothnagel s Spezielle Pathologie and Therapie, Wien 1900, vol. XIV, II, 2, page 322.

10. Dennig, A.: Ueber die Tuberkulose im Kindesalter, Leipzig 1896, page 193.

11. D'Espine et Picot, Deutsch von Ehrenhaus, Leipzig 1878, page 461.

12. Finkelstein, H.: Lehrbuch der Sáuglingskrankheiten Berlin 1905, I, 2.

13. Hennig, C.: Lehrbuch der Krankheiten des Kindes, Leipzig 1889, 2nd edition, page 272.

14. Henoch, E.: Vorlesungen über Kinderkrankheiten, Berlin 1892, 6th edition, page 409.

15. Holt, E. L.: The diseases of infancy and childhood, New York 1911, 6th edition, page 1039.

16. Hutinel, V.: Les Maladies des Enfants, Paris 1909, vol. IV, page 350.

17. v. Hüttenbrenner, A.: Lehrbuch der Kinderheilkunde, Wien 1876, page 302.

18. Jacobi, A.: Phthisis in Keating's Cyclopedia of the Diseases of Children, Philadelphia 1890, vol II, page 677.

19. Koplik, H.: The Diseases of Infancy and Childhood, New York and Philadelphia, 1902, page 246.

20. v. Pirquet, C.: Tuberkulose in Feer s Lehrbuch der Kinderheilkunde, 2nd edition, Jena 1912, page 695.

21. Rotch, T. M.: Pediatrics, Philadelphia 1901, page 396.

22. Steiner, J.: Compendium der Kinderkrankheiten, 2nd edition, Leipzig 1873, page 181.

23. Sticker, G.: Lungenblutungen, in Nothnagel's Spezielle Pathologie and Therapie, Wien 1900, vol. IV, page 57.

24. Thomson, J.: Guide to the Clinical Examination and Treatment of Sick Children, Edinburgh and London 1908, 2nd edition, page 264.

25. Unger, L.: Lehrbuch der Kinderkrankheiten, Leipzig and Wien 1894, 2nd edition, page 230.

26. West, Chas.: Pathologie and Therapie der Kinderkrankheiten, Deutsch von Honoch, Berlin 1872, 5th edition, page 282.

27. Young, R. A.: in Kelynack's Tuberculosis by various writers, London 1908, page 85.

28. Zuber: Tuberculose Pulmonaire, in Grancher, Comby et Marfan, Traité des maladies de l'enfance, Paris 1904, vol. IV, page 206.

29. Rasmussen, V.: Hospitals Tidende, 1872, page 109 and 113.

30. Hinz, E. R.: Ueber profuse Haemoptoe im frühen Kindersalter bei der Lungentuberkulose, Inaugural Dissertation, Leipzig 1903.

31. Aldibert, A.: Deux cases d'adénopathie trachéobronchique avec hémoptysies foudroyantes, Revue mensuelle de l'enfance 1891.

32. Barety, A.: Adénopathie trachéobronchique, These de Paris 1874.

33. Barthez et Sanné: Traité clinique et pratique des maladies des enfants.

34. Baumel, L.: Précis des maladies des enfants, Paris 1904, page 398.

35. Becquerel: Traité théorique et pratique des maladies des enfants 1842.

36. Berton, A.: Traité des maladies des enfants, Paris 1837.

37. Berton, A.: Traité pratique des maladies des enfants, Paris 1842.

38. Bouchut: Traité pratique des maladies des nouveau-nés et des enfants a la mamelle, 1867.

39. Bulius, W.: Zur Klinik und Diagnostik der Tuberkulose im ersten Lebenjahr, Jahrbuch für Kinderheilkunde 1899, vol. 49, page 304.

40. Cadet de Gassicourt: Journal de médicine de Paris, 1886, March 21.

41. Carrié, M.:Hémoptysie foudroyante chez un enfant de deux ans et demi, Gazette médicale de Paris No. 18.

42. Cumston, C. G.: Remarks on Certain Accidents Occurring in Pulmonary Tuberculosis in Childhood, 1894.

43. Foss, R. W.: British Medical Journal, August 1879, page 171.

44. Fronz, E.: Beitrag zur Lehre von der Bronchialdrüsentuberkulose, Jahrbuch für Kinderheilkunde 1897, vol. 44.

45. Henoch, E.: Handbuch der Kinderkrankheiten, 1895, 7th edition.

46. Hertz: In v. Ziemssen Hanbuch der speziellen Pathologie und Therapie, vol. V, page 288.

47. Hoffnung, J.: Über Haemoptoe bei Kindern, Inaugural Dissertation, Berlin 1885.

48. Hohlfeld, M.: Zur tuberculősen Lungenphthise im Sáuglingsalter, Münchener Medizinische Wochenschrift, 1902, November 25th.

49. Howard: Quoted in Gerhardt und Seifert.

50. Kessel, H.: Die Tuberkulose im frühen Kindesalter, Archiv für Hygiene, 1895, vol. 21.

51. Lebert: Klinik der Brustkrankheiten, Tübingen 1879.

52. Mantel, P.: Hémorrhagies tuberculeuses d'origine intrapulmonaire chez les enfants au-dessous de 7 ans, Le progres médicale 1886.

53. Meusnier, F. E.: Des Hémoptysies chez les enfants, Thése de Paris 1892.

54. Michael, I.: Über einige Eigenthümlichkeiten der Lungentuberkulose, Jahrbuch für Kinderheilkunde 1884, vol. 22.

55. Peter, M.: Hémoptysie tuberculeux et phthisis ab haemoptoe, L'Union médicale, 1870, No. 36.

56. Powell, D. R.: British Medical Journal, May 30th, 1874.

57. Powell, D. R.: The Lancet, December 1st, 1875.

58. Rasmussen, V.: Nord; med. Archiv, vol. I, No. 12.

59. Revillied, E.: Hémoptysie, Thése de Paris 1886.

60. Steffen, A.: Klinik der Kinderkrankheiten, 1865.

61. Steffen, W.: Jahrbuch für Kinderheilkunde, 1894, vol. 38, page 377.

62. Widerhöfer, W.: Die Erkrankungen der Bronchialdrüsen, in Gerhardt's Handbuch, vol III, part 2, page 988.

63. Ausset: Sur un cas d'hémoptysie mortelle chez un enfant de 8 ans, Gazette hebdom. 1899, No. 22.

64. Bierbaum, J.: Erlebnisse aus der Kinderpraxis, Journal für Kinderkrankheiten, 1868, Heft 1 and 2.

65. Ganjoux et Maillet: Tuberculose polyviscérale chez l'enfant, Annales de médicine et chirurgie infantile, 1911, vol. 15, page 291.

## Figures and Tables

**Figure f1:**
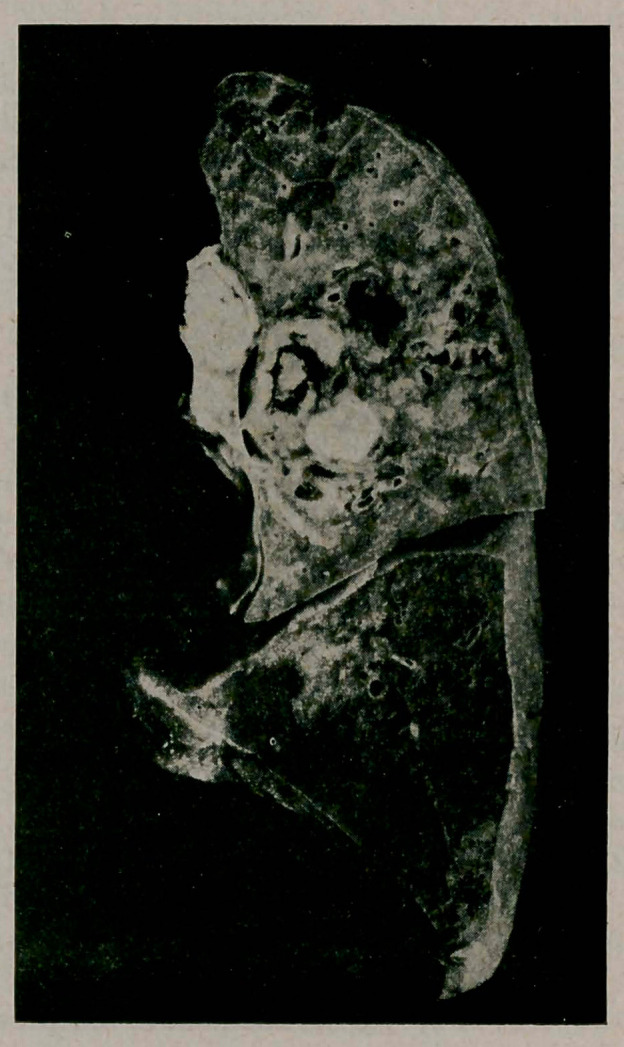


**Figure f2:**